# Physical fitness disparities among New York City public school youth using standardized methods, 2006-2017

**DOI:** 10.1371/journal.pone.0227185

**Published:** 2020-04-09

**Authors:** Kevin J. Konty, Sophia E. Day, Michael Larkin, Hannah R. Thompson, Emily M. D’Agostino

**Affiliations:** 1 NYC Department of Health and Mental Hygiene, Office of School Health, New York City, New York, United States of America; 2 Department of Epidemiology and Biostatistics, Graduate School of Public Health and Heath Policy, City University of New York, New York City, New York, United States of America; 3 Learning, Teaching and Assessment, Plainedge School District, Massapequa, NY, United States of America; 4 Department of Community Health Sciences, School of Public Health, University of California Berkley, Berkeley, California, United States of America; 5 Department of Family Medicine and Community Health, Duke University School of Medicine, Durham, North Carolina, United States of America; Leibniz Institute for Prevention Research and Epidemiology BIPS, GERMANY

## Abstract

Standardized physical fitness monitoring provides a more accurate proxy for youth health when compared with physical activity. Little is known about the utilization of broad-scale individual-level youth physical fitness testing to explore health disparities. We examined longitudinal trends in population-level fitness for 4^th^-12^th^ grade New York City youth during 2006/7-2016/17 (average n = 510,293 per year). Analyses were performed in 2019. The primary outcome was whether or not youth achieved sex-/age-specific performance levels (called the Healthy Fitness Zone) on the aerobic capacity, muscular strength and muscular endurance tests using the NYC FITNESSGRAM. The Cooper Institute’s most recent Healthy Fitness Zone criteria were applied to all tests and years. Prevalence estimates were weighted, accounted for school clustering, adjusted for student-level sociodemographics, and run by sociodemographic subgroups and year. The overall prevalence for meeting 3 Healthy Fitness Zones increased from 15.5% (95%CI: 13.9%-17.0%) in 2006/7 to 23.3% (95%CI: 22.2%-24.4%) in 2016/17 for students in grades 4–12. Fitness for all student groups increased over time, although Hispanic and non-Hispanic black girls consistently had the lowest prevalence of meeting 3 Healthy Fitness Zones as compared to all other race/sex subgroups. Also, 9^th^-12^th^ graders had a lower prevalence of meeting 3 Healthy Fitness Zones as compared to 4^th-^8^th^ graders. Given forecasted sharp increases in cardiovascular disease prevalence, routine youth fitness surveillance using standardized, criterion referenced methods can identify important fitness disparities and inform interventions.

## Introduction

Just 25% of United States (US) youth are reported to meet national physical activity guidelines including at least 60 minutes of daily physical activity for children ages 6 and older [1]. Low youth physical activity levels are of particular concern given a large body of research demonstrating the benefits of physical activity for children’s health [2]. For example, youth physical activity interventions are shown to reduce blood pressure, promote a healthier lipid profile, reduce psychosocial stress and depression, and decrease risk of metabolic syndrome [2–5]. Low youth physical activity also corresponds to low physical fitness, including near failing grades on criterion-referenced health-related fitness standards for US children and adolescents [6,7]. Physical fitness can be defined as a state reflecting one’s ability to perform physical activity or exercise that is related to both present and future health [8,9]. Reduced fitness is strongly correlated with noncommunicable chronic conditions in childhood and adulthood, including cardiovascular disease, type 2 diabetes, metabolic syndrome, dementia and Alzheimer disease, breast and colon cancer, and disability later in life [10]. Moreover, disparities persist in youth fitness attainment across race, poverty, and sex [11–13], therein predicting persistent inequities for chronic conditions into adulthood [14].

Recent research has expanded on the utilization of health-related fitness testing in school-aged youth as a population-level surveillance initiative. This work aims to provide timely information about patterns and trends to inform scientific research, physical activity programming, and clinical arenas [15–17]. The concept of health-related fitness was first introduced by the US Task Force on Youth Fitness in 1977, which demonstrated a strong relationship between physical fitness and health. The FitnessGram® is the most widely used criterion-referenced health-related fitness test, developed by the Cooper Institute for Aerobics Research and administered to youth globally in school and research settings [9]. The five components assessed by the FitnessGram® include cardiorespiratory fitness, body composition, muscular strength, muscular endurance, and flexibility.

Prior work has shown the effectiveness of physical activity level surveillance in youth and its importance for informing programs and policies targeting reduced burden of non-communicable diseases [18–21]. However, other reports have shown that few physical activity monitoring studies draw from large samples and standardized, criterion-referenced measures at the individual-child level to accurately assess population trends [22]. Specifically, monitoring fitness using standardized measurement methods provides a more accurate proxy for child and adolescent health when compared with physical activity monitoring [23,24]. However, the Cooper Institute has re-established measurement and criteria for fitness attainment four times over the last two decades [25] complicating the tracking of fitness over time across unstandardized measures. Moreover, little is known about the utilization of broad-scale individual-level youth physical fitness testing to explore health disparities.

The purpose of this study was to provide longitudinal trends and sociodemographic differences in population-level physical fitness for New York City (NYC) public school youth in grades 4–12 during the 2006/7-2016/17 school years using individual, child-level data drawn from standardized health-related fitness assessments. To our knowledge, this is the first paper to report physical fitness trends and sociodemographic patterns in a single large urban school district. The underlying data are longitudinal and measured annually for all students, allowing for characterization of year-to-year changes within the same student population. Findings from this work can contribute to a growing literature that supports youth fitness surveillance efforts to identify potential wide-scale fitness disparities and inform population-level youth health interventions.

## Materials and methods

This study was approved by the City University of New York Institutional Review Board (IRB File #2015–0582) and the DOHMH Institutional Review Board (Protocol # 14–019) and was determined by these boards to be public health surveillance that is not research and therefore exempt from the requirement for obtaining written informed consent. Data were drawn from the NYC FITNESSGRAM dataset, NYC Department of Education (DOE)’s citywide fitness assessment based on the FitnessGram®, and jointly managed by NYC DOE and Department of Health and Mental Hygiene (DOHMH). NYC FITNESSGRAM comprises annual fitness assessments collected by NYC DOE for approximately 860,000 public school students per year starting in 2006/7.

The NYC FITNESSGRAM test is conducted annually by physical education and classroom teachers with students in 4^th^– 12^th^ grade and consists of 5 assessments designed to measure distinct components of health-related fitness. Aerobic capacity is evaluated using estimates of VO_2_max (maximal oxygen uptake), which reflects the maximum rate at which the cardiovascular, respiratory, and muscular systems take in, transport, and use oxygen during physical activity. VO_2_max is assessed by the PACER Test (the Progressive Aerobic Cardiovascular Endurance Run), in which students complete as many shuttle runs as possible back and forth across a 15-meter course in time to an audio recording that is paced to get faster every minute [9]. Body composition is measured by body mass index (BMI). Muscular strength and muscular endurance are measured via push-ups (performed at a 90° elbow angle and conducted at a specified pace until a student cannot complete any more) and curl-ups (i.e., sit-ups, conducted with knees flexed and feet free, also performed to a specified pace with students completing as many as possible). Flexibility is assessed by the sit and reach. This test battery is demonstrated to have both strong reliability and validity [9,24].

Consistent with prior analyses of longitudinal NYC FITNESSGRAM data and assessments of youth health-related fitness levels [26–28], this study reports on PACER, curl-up and push-up test data, which are valid and reliable measures of aerobic capacity, abdominal and upper body strength and endurance, respectively [9]. These 3 tests have been shown to predict cardiovascular disease risk and adiposity, and are the basis for recommendations for health monitoring systems to include youth fitness testing [9]. BMI is not assessed using physical ability tests, unlike aerobic capacity, muscular strength and muscular endurance. Although body composition is also related to fitness [29–31], it cannot be characterized as a fitness test, and has thus been excluded from this analysis. Flexibility has also been excluded, due to the lack of consistent evidence on its association with youth fitness [32,33].

Students were included in this study if they were: 1) enrolled in a general education (i.e., non-charter or special education) NYC public school during 2006/7-2016/17; 2) aged 9–19 years as of December 31^st^ of the school year and in grades 4–12; and 3) had non-missing PACER, curl-up and push-up data in the given school year.

### Primary outcome

The primary outcome was a binary variable representing whether the student met the performance criteria for the Cooper Institute’s sex- and age-specific Healthy Fitness Zones (HFZ) for all three tests. The sex- and age-specific HFZ indicate whether a student met criterion-referenced physical fitness levels on each test, and provides an indication of present and future health [34,35]. We standardized HFZ criteria for all PACER, push-up, and curl-up tests and years of testing (2006/7-2016/17) based on the Cooper Institute’s most current HFZ criteria [9], and examined students who met the criteria for different total number of HFZ: (1) 3 HFZ vs. ≤2 HFZ, (2) ≥2 HFZ vs. ≤1 HFZ, and (3) ≥1 vs. 0 HFZ.

### Covariates

Multiple factors known to be associated with youth fitness [6] were included as covariates or potential effect modifiers. Time-varying student-level variables included age, individual student household poverty, and student’s home neighborhood socioeconomic status. Individual student household poverty was based on student eligibility/non-eligibility for free/reduced price school meals through the National School Lunch Program which provides meal assistance according to household income at or below 185% of the federal poverty level [36]. Students’ home addresses were geocoded to determine the students’ home census tract for each school year. Missing home addresses were imputed using addresses from data recorded for the same child in other years. Consistent with DOHMH guidelines [37], a child’s home neighborhood’s socioeconomic status was defined according to American Community Survey 2008–2012 data as the percentage of households in the students’ home census tract living below the federal poverty threshold (low [<10%], medium [10%-20%], high [>20%-30%], and very high [>30%] area poverty) and defined according to the Census 2010 boundaries [38].

Fixed (i.e. non time-varying) student-level variables included grade (4^th^– 12^th^); sex (male/female); race (Hispanic, non-Hispanic black, non-Hispanic white, Asian/Pacific Islander, and other/multiple races); place of birth (US or foreign born); and language spoken at home (English, Spanish or other). Fixed school-level variables included the school borough by neighborhood health action center status. These centers are Health Department buildings that co-locate health services, community health centers, public hospital clinical services, community-based organizations and service providers [39]. Missing or discrepant values for sociodemographic variables were resolved, when possible, using information for the same student from other years.

### Statistical methods

Observations with complete sex, date of birth, PACER, push-up, and curl-up assessments were weighted to be representative of the NYC public school 4^th^-12^th^ grade enrollment population for each 2006/7-2016/17 school year, accounting for individual- and school-level characteristics. For each school year and grade type (elementary, middle, high school), observations with completed assessments were weighted using standard raking procedures to match marginal control totals for: age, race by neighborhood (defined as borough by neighborhood heath action center area), grade by sex by neighborhood, and individual student household poverty by neighborhood. This is very similar to post-stratification for non-random survey nonresponse. Additional details are described elsewhere [40,41]. Descriptive statistics were computed to summarize sample characteristics.

Prevalence estimates for students meeting 3 HFZ criteria were examined over time and across sociodemographic variables including student grade, sex, race, home neighborhood socioeconomic status and home borough. Prevalence rates were also calculated over time for students meeting (1) 3 HFZ vs. ≤2 HFZ, (2) ≥2 HFZ vs. ≤1 HFZ, and (3) ≥1 vs. 0 HFZ and applying the same marginal control totals listed above.

Spatial analyses were conducted using Census 2010 boundaries to assess student fitness prevalence in 2016–17 across home borough based on students’ geocoded home addresses. Estimates were also calculated over time for sex by race subgroups. Additional estimates were generated over time for students stratified by grade level groups (4^th^-8^th^ grades vs. 9-12^th^ grades) based on prior literature documenting sharp drop-offs in physical activity in adolescence [20,42].

To examine longitudinal trends in population-level fitness, a logistic regression model for each of the 3 HFZ categories (i.e., (1) 3 HFZ vs. ≤2 HFZ, (2) ≥2 HFZ vs. ≤1 HFZ, and (3) ≥1 vs. 0 HFZ) was built where the probability of being in a given category was modeled on time (an integer value that increases from 0 to 10 corresponding to the 2006/7 to 2016/17 school years). Standard errors were clustered at the school- and student-level, and models were adjusted for student age, grade level, sex, race, language spoken at home, place of birth, individual student household poverty, and the school borough by neighborhood health action center neighborhood status. Similar logistic models stratified by student grade type, sex, race, place of birth, home language, and home neighborhood socioeconomic status were performed using logistic models were also ran.

All statistical analyses were performed in 2019 using SAS 9.4 software (Cary, NC).

## Results

On average, 78% of students in grades 4–12 who were eligible to have FITNESSGRAM measurements completed all three fitness assessments from 2006/7-2016/17 (n = 5,613,228 total; mean 510,293 students per year). Sample sizes for sociodemographic and fitness subgroups are included in Table 1.

**Table 1:** Sociodemographic and Healthy Fitness Zone characteristics of New York City public school students, grades 4–12 (unweighted n = 567,461^a^ and weighted n = 646,201^b^) 2016/17.

	Unweighted n	%	Weighted n	%
Sex				
Female	278506	49.1%	315938	48.9%
Male	288955	50.9%	330272	51.1%
Grade Level				
Elementary/Middle School (4–8)	325207	57.3%	353119	54.6%
High School (9–12)	242254	42.7%	293091	45.4%
Race				
Hispanic	226845	40.0%	261999	40.5%
Non-Hispanic black	136009	24.0%	159878	24.7%
Asian and/or Pacific Islander	106527	18.8%	116008	18.0%
Non-Hispanic white	88765	15.6%	97786	15.1%
Other^c^	9315	1.6%	10539	1.6%
Language Spoken at Home				
English	306789	54.1%	349241	54.0%
Spanish	141494	24.9%	164497	25.5%
Other language	119058	21.0%	132312	20.5%
Place of Birth				
US	454552	80.1%	512798	79.4%
Foreign	112532	19.8%	132990	20.6%
Healthy Fitness Zones Met^d^				
3	135479	23.9%	150518	23.3%
2	194028	34.2%	220710	34.2%
1	150021	26.4%	172562	26.7%
0	87933	15.5%	102420	15.8%
Home Neighborhood Poverty^e^				
0% to <10%	120475	21.2%	133220	20.6%
10% to 20%	160047	28.2%	179742	27.8%
>20% to 30%	135629	23.9%	155573	24.1%
>30% to 100%	149358	26.3%	175371	27.1%
Free/Reduced Meal Status^f^				
Free/reduced meals	405837	71.5%	462757	71.6%
Full-price meals	161624	28.5%	183453	28.4%

^a^Total unweighted n (2006/7-2016/17) = 5,613,228

^b^Total weighted n (2006/7-2016/17) = 7,252,490

^c^Other race includes Native American and multiple races

^d^Based on whether the student met the performance criteria for the Cooper Institute’s most recent sex- and age-specific Healthy Fitness Zones for all three tests [9]

^e^Neighborhood socioeconomic status was defined according to American Community Survey 2008–2012 data as the percentage of households in the students’ home census tract living below the federal poverty threshold (low [<10%], medium [10%-20%], high [>20%-30%], and very high [>30%] area poverty) and defined according to the Census 2010 boundaries [38]

^f^Individual student household poverty (high vs. low) was based on student eligibility/non-eligibility for free/reduced price school meals through the National School Lunch Program which provides meal assistance according to household income at or below 185% of the federal poverty level [36].

**Fig 1:**
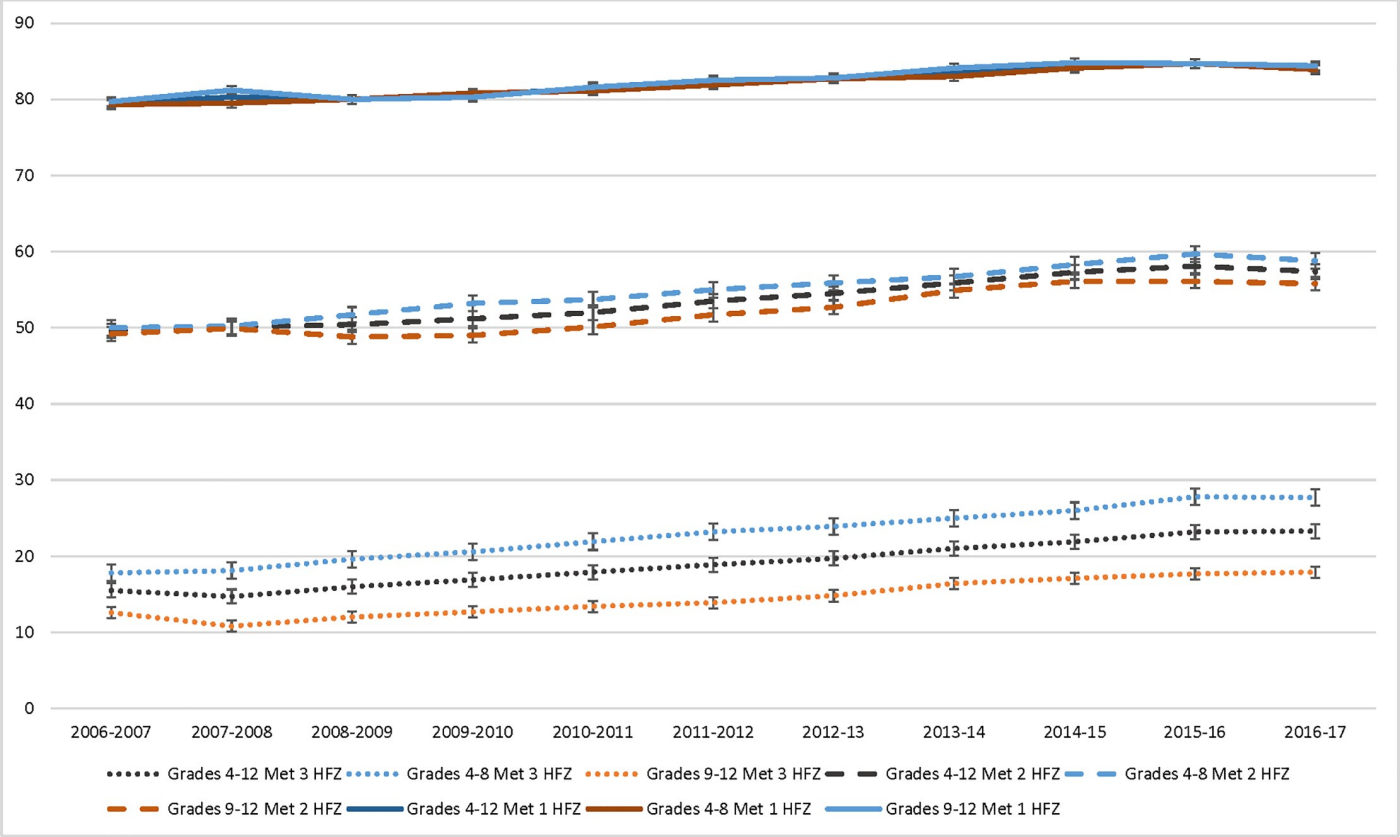
Percentage of New York City public school students grades 4–12 who met three, two and one Healthy Fitness Zone(s), overall and by grade level, 2006/7-2016/17.

**Fig 2:**
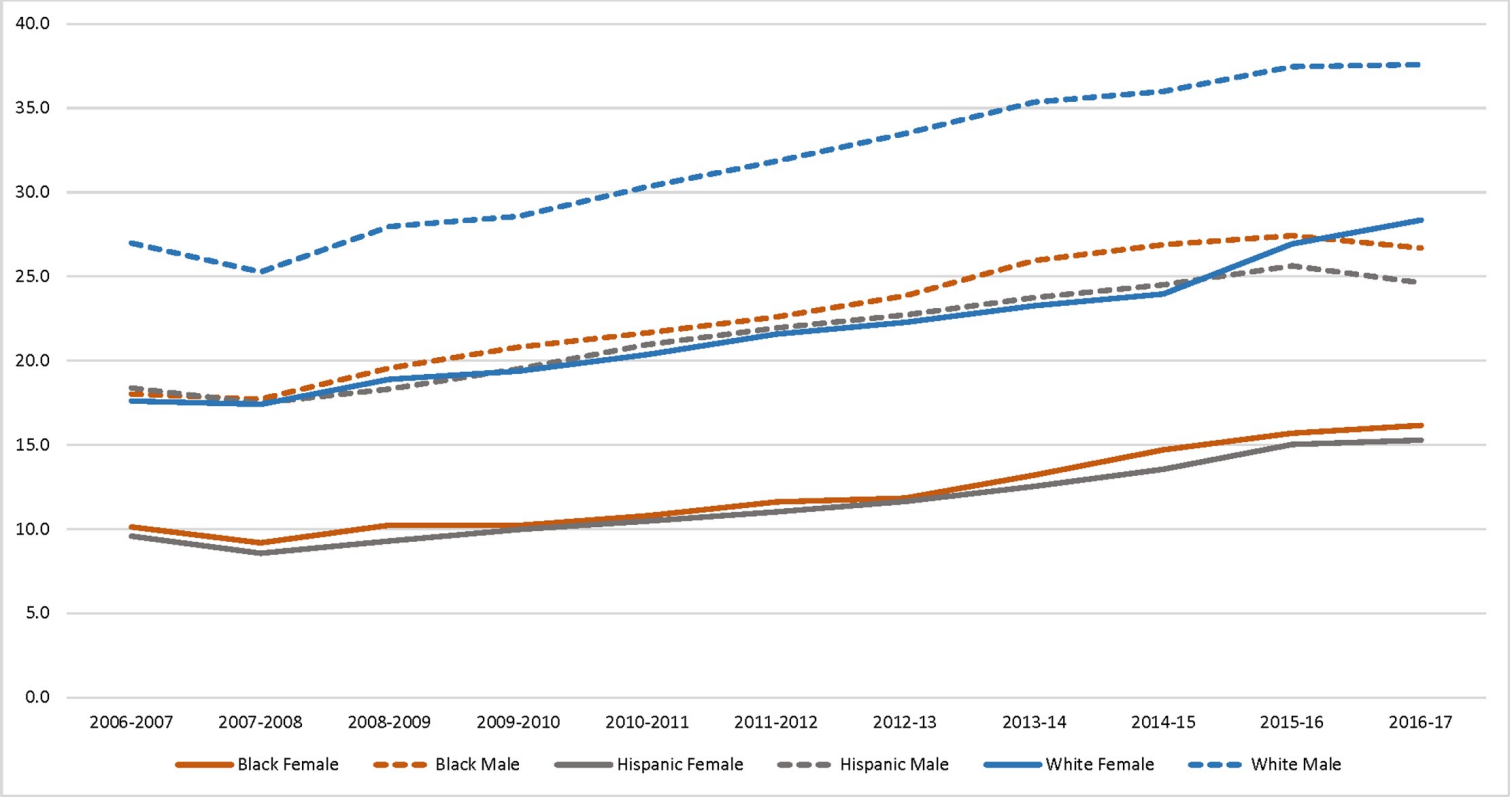
Percentage of New York City public school students grades 4–12 who met three, two and one Healthy Fitness Zone(s), across race and sex, 2006/7-2016/17.

Weighted estimates for percent of students who met HFZ criteria across time by grade, sex, and race appear in S1 Table and Figs 1, 2 and S1. Over time, prevalence of students who met 3 HFZ increased in all subgroups, although gaps widened across all sociodemographic factors. The overall prevalence for meeting 3 HFZ (i.e., the highest level of fitness) increased from 15.5% (95%CI: 13.9%-17.0%) in 2006/7 to 23.3% (95%CI: 22.2%-24.4%) in 2016/17 for all students in grades 4–12. The greatest growth in meeting 3 HFZ was shown in 4^th^-8^th^ grade students (17.8% [95%CI: 16.3%-19.3%] in 2006/7 to 27.7% [95%CI: 26.3%-29.1%] in 2016/17; Figs 1 and S1).

Across all grade levels, increases were found over time for students who met all HFZ categories (1 HFZ [e.g., low fitness], 2 HFZ [e.g. mid fitness], and 3 HFZ [e.g. highest fitness]). However, disparities increased over time across grade level subgroups as the number of HFZ achieved increased, including a 9.9% vs. 5.3% increase from 2006/7 to 2016/17 for students in grades 4–8 vs. 9–12, respectively, meeting 3 HFZ, compared with 8.8% vs. 6.6% meeting 2 HFZ, and 4.6% vs. 4.7% meeting 1 HFZ (S1 Table and Figs 1 and S1).

Across sex, boys improved more in HFZ attainment over time (19.7% [95%CI: 17.7%-21.6%] to 27.9% [95%CI: 26.8%-29.1%], from 2006/7-2016/17) compared with girls (11.2% [95%CI: 9.8–12.5] to 18.5% [95%CI: 17.4%-19.6%], from 2006/7-2016/17; S1 Table). Gaps were also large across race (22.5% [95%CI: 19.4%-25.6%], 14.0% [95%CI: 12.3%-15.7%] and 14.0% [95%CI: 12.4%-15.6%] for non-Hispanic white, non-Hispanic black and Hispanic students in 2006/7 vs. 33.1% [95%CI: 30.5%-35.7%], 21.4% [95%CI: 20.4%-22.5%] and 20.1% [95%CI: 19.1%-21.0%] for non-Hispanic white, non-Hispanic black and Hispanic students in 2016/17, respectively; S1 Table).

Across race and sex, all subgroups increased in fitness over time, although Hispanic and non-Hispanic black girls consistently had the lowest prevalence for meeting 3 HFZ, and race/sex gaps widened (Fig 2 and S2 Table). For example, in 2006/7, the prevalence for achieving 3 HFZ was 9.6%, (95%CI: 8.3%-10.8%) in Hispanic girls and 10.1% (95%CI: 8.7%-11.5%) in non-Hispanic black girls vs. 17.6% (95%CI: 14.3%-20.9%) in white girls and 27.0% (95%CI: 23.8%-30.1%) in white boys. By comparison, in 2016/17, 15.3% (95%CI: 14.3%-16.2%) of Hispanic girls and 16.2% (95%CI: 15.1%-17.2%) of non-Hispanic black girls, vs. 28.4% (95%CI: 25.6%-31.1%) of white girls and 37.6% (95%CI: 34.9%-40.2%) of white boys met 3 HFZ criteria.

**Fig 3:**
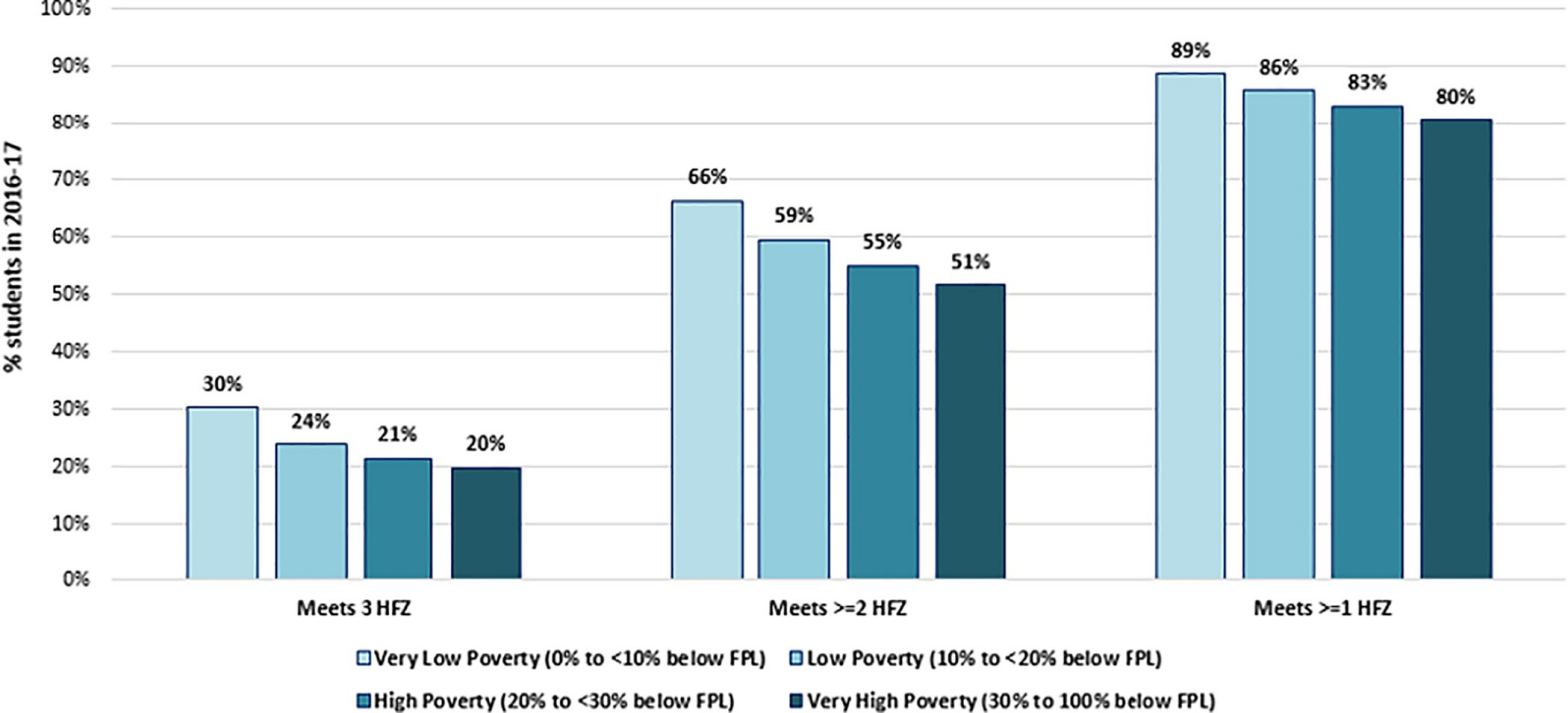
Percentage of New York City public school students, grades 4–12, who met three, two and one Healthy Fitness Zone(s) across student home neighborhood poverty level, 2016/17.

Overall patterns by home neighborhood socioeconomic status showed that as home neighborhood poverty decreased, the percent of students meeting HFZ criteria increased (Fig 3). For example, 30.1% (95%CI 28.0% to 32.1%) vs. 19.7% (95%CI 18.7% to 20.7%) of students living in neighborhoods with low compared with high area poverty met HFZ criteria in 2016/17.

**Fig 4:**
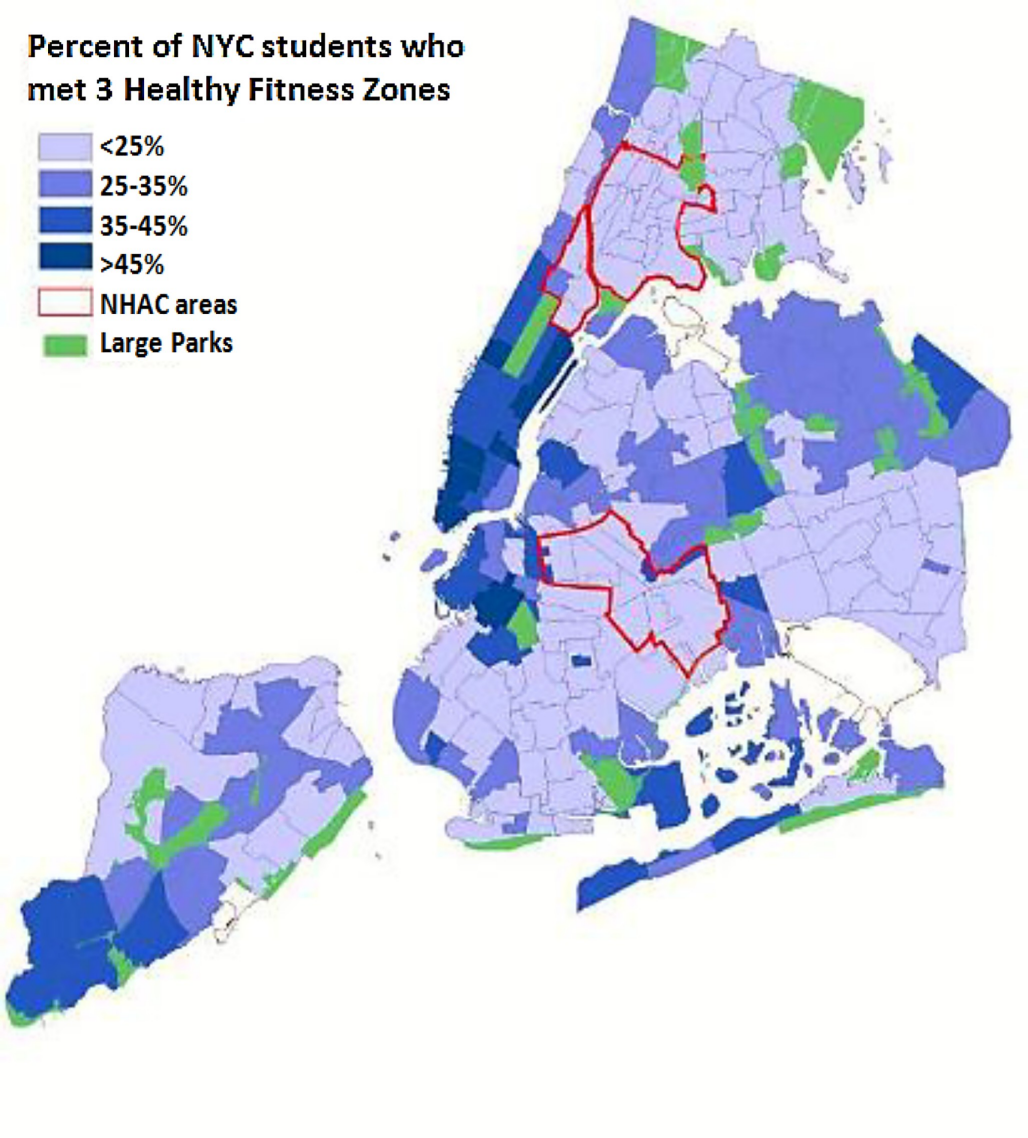
Geographic distribution of percentage of public school students, grades 4–12, who met three Healthy Fitness Zones across New York City, 2006/7-2016/17.

Spatial analyses showed that disparities in achieving 3 HFZ widened from 2006 to 2017 across home borough (from 13.7% [95%CI: 10.8%-16.6%] to 22.5% [95%CI: 20.6%-24.4%] in the Bronx vs. 24% [95%CI: 18.6%-29.3%] to 39.6% [95%CI 35.6%-43.5%] in Manhattan. The lowest fitness levels were observed in the poorest areas of NYC (i.e., NHAC areas; Fig 4).

Analyses of longitudinal trends (Figs 1 and 2) showed significant differences within grade type, sex, race, place of birth, and residential poverty (all p < .001) and for language spoken at home (p<0.01).

## Discussion

This study found that just under a quarter of NYC public school 4^th^-12^th^ grade students in 2017 met the criteria for health-related fitness on 3 standardized fitness tests of aerobic capacity, muscular strength and muscular endurance. Findings from this study also showed a significant increasing trend in prevalence of New York City public school students across grades 4–12 from 2006/7 to 2016/17 who met HFZ criteria. However, our findings demonstrate a significant widening in sociodemographic disparities in fitness across student grade, sex, race and poverty over time. Specifically, we found large disparities in fitness attainment in girls, non-Hispanic black and Hispanic youth, youth living in high poverty, and older students. Given the burden of widening health inequities in combination with forecasted sharp increases in the prevalence of cardiovascular disease [7], surveillance of youth fitness might suggest the origin of later population-level fitness disparities and the need for tailored interventions for high-need subgroups, preferably at younger ages.

Findings here correspond with the literature demonstrating large declines in physical activity from childhood to adolescence [20,42–45]. For example, Welk et al. showed declines in Hungarian youth meeting the HFZ for aerobic capacity with increasing age from 10–18 years [44]. Ortega et al. showed increased sedentary time and reduced moderate to vigorous physical activity from childhood to adolescence (ages 9–15) in Estonian and Swedish youth [45]. These trends are also found in NYC, where 40% and 20% of youth ages 6–12 and 14–18, respectively, meet physical activity recommendations [46,47].

Sex-related disparities in youth fitness also have been shown. Marta et al. found that boys demonstrated higher aerobic fitness, speed, and strength and girls had higher flexibility scores [48]. Flanagan et al. similarly found that boys’ cardiorespiratory endurance and power performance was higher than for girls in both the fourth and fifth grades [49]. Moreover, Messiah et al. found that boys aged 9–16 had significantly higher fitness scores compared with girls for the PACER, sit-up and push-up tests [50].

Similarly, fitness disparities across race are also well-documented. Katzmarzyk et al. found lower fitness in non-Hispanic black and Hispanic/Mexican American vs. non-Hispanic white 12–15 year-old youth, respectively [6]. Currently, 8.6% of non-Hispanic black and 7.6% of Hispanic US youth ages 2–19 compared with 4.4% of their non-Hispanic white counterparts have severe obesity [51], defined as ≥120% of the 95^th^ percentile of body mass index adjusted for age and sex. Severe obesity in youth is associated with many cardiometabolic comorbidities [52] and poorer cardiometabolic risk profiles into adulthood [53].

The World Health Organization calls for affordable population-based prevention strategies for reducing the global burden of cardiovascular disease on morbidity and mortality [54]. Schools may hold a critical role in providing youth with safe and accessible physical activity [2]. However, less than 10% of US schools provide daily physical education to their students in all grades, and only 57% of US school districts require regularly scheduled recess for elementary school students [55]. Moreover, school-based programming in the US is insufficient to support youth in meeting daily physical activity recommendations in and of itself [56,57]. Expanding and supporting opportunities for school-based physical activity, specifically through evidence-based approaches to increasing youth physical activity and fitness, is necessary to help youth meet these guidelines.

In light of our findings, youth physical activity initiatives are still needed to improve health-related fitness levels at a population level. School-based interventions to increase physical activity have shown short-term effectiveness in improving youth fitness levels [58–61], such as through offering intensive physical activity curriculums (e.g., football, interval training and strength training) in physical education classes [58,59], individually tailored physical exercise [60] and playground redesigns [61]. However, findings are mixed in regard to the long-term (three-seven year) effects of school-based physical activity interventions [62]. Alternatively, some studies have proposed population-wide youth fitness promotion through active school transport (walking and cycling) [63], community-school organization collaborations (such as community-school marathon programs) [64], park-based afterschool programs [12], and community fitness facility programs with active youth recruitment [65]. Others have suggested the need for engaging physical education teachers in the design of school-based fitness promotion efforts [66].

In NYC, physical activity and fitness promotion programs include the DOE’s Office of School Wellness Programs which offers the Move-to-Improve and Cooperative, Healthy, Active, Motivated, Positive Students, HealthCorps initiatives, and PE Works [67–70]. The Move-to-Improve program is a classroom-based physical education program for K-3^rd^ grade teachers to integrate core academic requirements into fitness breaks [67]. Similarly, Cooperative, Healthy, Active, Motivated, Positive Students provides NYC middle schools with sports and fitness program resources for students outside of school hours [68]. HealthCorps, a NYC school-based peer mentorship program for high school students reported a 45% increase in teen physical activity levels after one year of participation in a year-long physical fitness and active lifestyle discussion and activity-based curriculum [69]. PE Works is a multi-year DOE improvement plan to address known physical activity barriers and revitalize Physical Education for K-6^th^ grade students [70]. These programs seek to promote positive attitudes towards exercise, and increased opportunities for physical activity engagement in NYC youth, therein setting up healthy behavior patterns that may serve to reduce fitness disparities into adulthood. Future analyses will examine the impact of these programs on trends in student fitness.

### Strengths and limitations

Strengths of this analysis include a population-level analysis of health-related fitness prevalence across all of NYC public school youth in grades 4–12 (representing a total of n = 7,252,490 observations) over an 11-year study period and drawing from criterion-referenced individual-level youth fitness measurements. The NYC public school system is the largest in the country, serving 1.1 million children in nearly 1,700 schools, and is among the most racially and socioeconomically diverse. Also, analyses from this study uniquely draw from individual-level longitudinal data that standardize fitness performance scores during shifts over time in measurement and reporting criteria. Although some information is available on changes in fitness across youth in other settings that administer the FitnessGram® [71–73], these reports do not account for changes in reporting standards, present only aggregate-level findings, and combine fitness with obesity measures despite evidence indicating that body composition is conceptually distinct from fitness tests [9].

This study has several limitations. First, our results do not include students from schools that are not mandated to participate in the NYC FITNESSGRAM, including private, charter, and special education schools (approximately 18%, 10%, and 2% of NYC school-aged children, respectively). Because public school students are more likely to live in poverty and to be racial minorities, findings may not be generalized to the entire NYC youth population. Second, fitness data was missing for 23% of eligible students, although we addressed this by weighting the measured population to be representative of the enrolled population accounting for individual- and school-level characteristics. Finally, there is potential for systematic bias and differential measurement error given FITNESSGRAM data are not collected for research purposes. Potential sources of random measurement error and systematic bias include variation across FITNESSGRAM testing sites where school staff may vary in their testing protocol. While NYC physical education teachers receive formal training on conducting the test, and protocols are designed to enhance consistency across administers, such as with manuals, video-based training, site-visits, and the use of calibrated scales [9], classroom teachers who administer the test may not receive the same level of training.

## Conclusions

This study drew from standardized population-level data from 2006/7-2016/17 in NYC school-aged youth and found improving levels of fitness, yet widening disparities across race, sex, poverty, and grade level subgroups. In light of declines in school-based opportunities for physical activity, findings here suggest that continued monitoring of individual-level student fitness is needed. Moreover, tailored interventions should be offered to high-need subgroups to better address the widening disparities in fitness levels reported here.

## Supporting information

S1 Fig Percentage of New York City public school students, grades 4–12, who met three, two and one Healthy Fitness Zone by 4–12 grade level, 2006/7-2016/17. (TIF)

S1 Table Percentage of New York City public school students, grades 4–12 who met criteria for three Healthy Fitness Zones, 2006/7-2016/17 (n = 7,252,490). (DOCX)

S2 Table Percent of New York City public school students, grades 4–12, over time and across race-sex subgroups who met criteria for 3 Healthy Fitness Zones, 2006/7-2016/17 (n = 7,252,490). (DOCX)
