# Supplementary figures and images for: Physical fitness disparities among New York City public school youth using standardized methods, 2006-2017
Source: PLoS One. 2020 Apr 9;15(4):e0227185. doi: 10.1371/journal.pone.0227185 (PMC7144992; doi:10.1371/journal.pone.0227185)

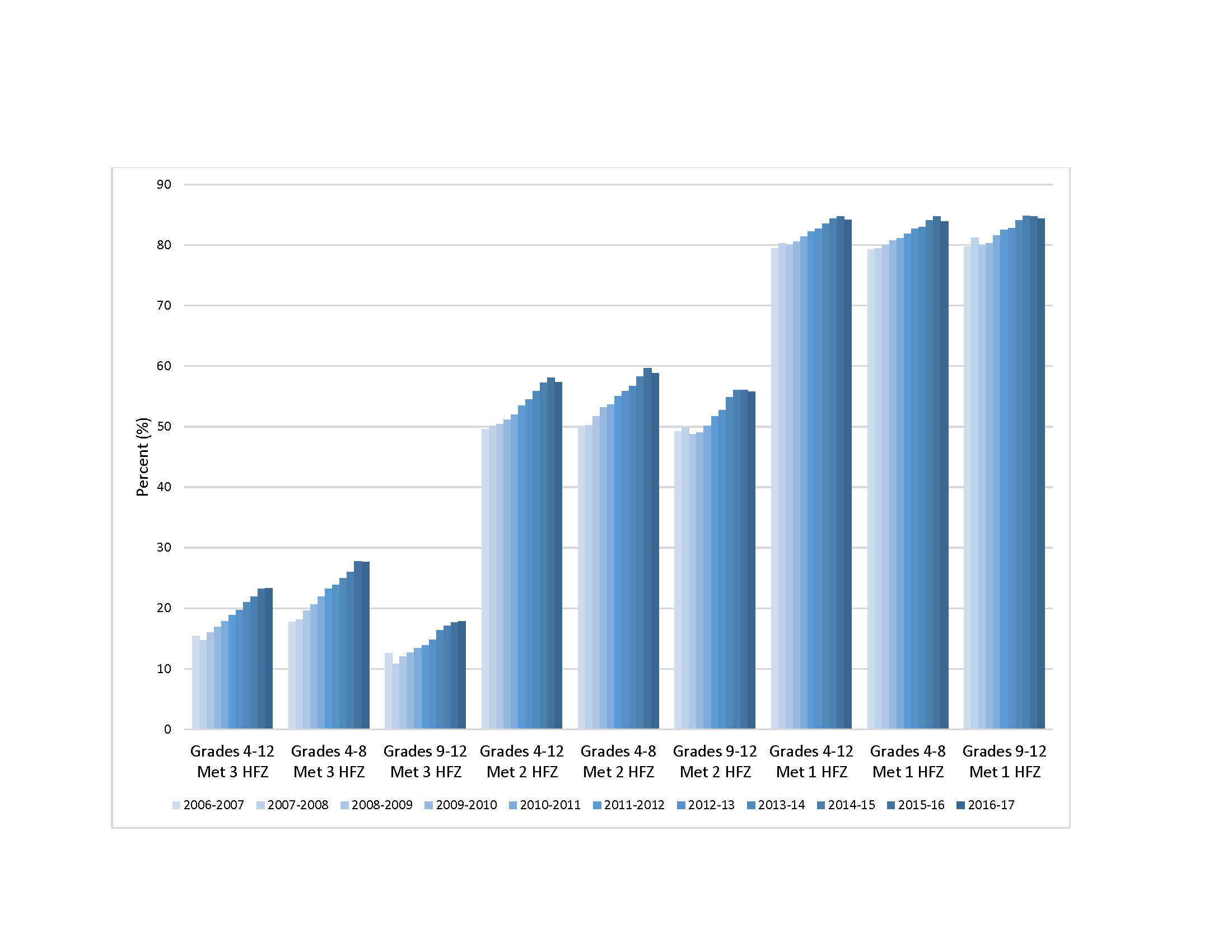

Supplement: S1 Fig — (TIF) [file pone.0227185.s001.tif]
