# Supplementary material for: Physical fitness disparities among New York City public school youth using standardized methods, 2006-2017
Source: PLoS One. 2020 Apr 9;15(4):e0227185. doi: 10.1371/journal.pone.0227185 (PMC7144992; doi:10.1371/journal.pone.0227185)
Supplement: S1 Table — (DOCX) [file pone.0227185.s002.docx]

|  | **Overall** | | | **Grades 4-8** | | | | **Grades 9-12** | | | | **Female** | | | | | | **Male** | | | | | | **Asian /Pacific** | | | | | | **Black** | | | | | | **Hispanic** | | | **White** | | | | |
| --- | --- | --- | --- | --- | --- | --- | --- | --- | --- | --- | --- | --- | --- | --- | --- | --- | --- | --- | --- | --- | --- | --- | --- | --- | --- | --- | --- | --- | --- | --- | --- | --- | --- | --- | --- | --- | --- | --- | --- | --- | --- | --- | --- |
| **Year** | % | 95% CI | | | % | 95% CI | | % | 95% CI | | | | % | | 95% CI | | | % | 95% CI | | | | % | | | 95% CI | | % | | | 95% CI | | | | % | | 95% CI | | % | | 95% CI | | |
| **2006/07** | 15.5 | 13.9 | 17.0 | 17.8 | | 16.3 | 19.3 | 12.6 | | 9.9 | 15.4 | | | 11.2 | | 9.8 | 12.5 | 19.7 | | 17.7 | 21.6 | 16.2 | | | 14.0 | | 18.4 | | 14.0 | | | 12.3 | 15.7 | 14.0 | | | 12.4 | 15.6 | | 22.5 | | 19.4 | 25.6 |
| **2007/08** | 14.7 | 13.5 | 15.9 | 18.1 | | 16.9 | 19.3 | 10.8 | | 8.9 | 12.7 | | | 10.4 | | 9.2 | 11.7 | 18.9 | | 17.5 | 20.2 | 16.0 | | | 14.2 | | 17.8 | | 13.4 | | | 12.2 | 14.6 | 13.1 | | | 11.9 | 14.3 | | 21.5 | | 18.8 | 24.1 |
| **2008/09** | 16.0 | 15.0 | 17.1 | 19.6 | | 18.3 | 20.8 | 12.0 | | 10.4 | 13.6 | | | 11.4 | | 10.4 | 12.5 | 20.5 | | 19.3 | 21.7 | 17.8 | | | 16.0 | | 19.5 | | 14.8 | | | 13.7 | 16.0 | 13.9 | | | 12.9 | 14.8 | | 23.6 | | 20.8 | 26.4 |
| **2009/10** | 16.9 | 15.9 | 18.0 | 20.6 | | 19.4 | 21.8 | 12.7 | | 11.0 | 14.4 | | | 11.9 | | 10.8 | 13.0 | 21.8 | | 20.6 | 23.0 | 19.1 | | | 17.2 | | 21.0 | | 15.4 | | | 14.4 | 16.5 | 14.8 | | | 13.9 | 15.7 | | 24.2 | | 21.3 | 27.1 |
| **2010/11** | 17.9 | 16.9 | 19.0 | 21.9 | | 20.7 | 23.1 | 13.4 | | 11.6 | 15.1 | | | 12.6 | | 11.4 | 13.7 | 23.2 | | 21.9 | 24.4 | 20.2 | | | 18.1 | | 22.3 | | 16.2 | | | 15.0 | 17.3 | 15.8 | | | 14.9 | 16.7 | | 25.6 | | 22.6 | 28.5 |
| **2011/12** | 18.9 | 17.8 | 20.0 | 23.2 | | 21.9 | 24.5 | 13.9 | | 12.2 | 15.7 | | | 13.4 | | 12.2 | 14.6 | 24.2 | | 23.0 | 25.4 | 21.1 | | | 19.0 | | 23.1 | | 17.1 | | | 15.9 | 18.3 | 16.6 | | | 15.6 | 17.5 | | 26.9 | | 23.9 | 30.0 |
| **2012/13** | 19.7 | 18.6 | 20.8 | 23.9 | | 22.6 | 25.2 | 14.8 | | 13.2 | 16.5 | | | 14.0 | | 12.8 | 15.2 | 25.3 | | 24.1 | 26.5 | 21.6 | | | 19.6 | | 23.7 | | 17.8 | | | 16.7 | 18.9 | 17.3 | | | 16.3 | 18.2 | | 28.1 | | 25.1 | 31.0 |
| **2013/14** | 21.0 | 20.0 | 22.1 | 25.0 | | 23.6 | 26.4 | 16.4 | | 14.9 | 17.8 | | | 15.1 | | 14.1 | 16.1 | 26.7 | | 25.6 | 27.9 | 22.7 | | | 20.7 | | 24.6 | | 19.6 | | | 18.5 | 20.6 | 18.3 | | | 17.3 | 19.2 | | 29.5 | | 26.8 | 32.2 |
| **2014/15** | 21.9 | 20.9 | 23.0 | 26.0 | | 24.7 | 27.4 | 17.1 | | 15.7 | 18.5 | | | 16.3 | | 15.2 | 17.3 | 27.4 | | 26.2 | 28.6 | 23.1 | | | 21.0 | | 25.1 | | 20.8 | | | 19.7 | 21.9 | 19.1 | | | 18.2 | 20.0 | | 30.2 | | 27.5 | 32.8 |
| **2015/16** | 23.2 | 22.1 | 24.3 | 27.8 | | 26.4 | 29.2 | 17.7 | | 16.3 | 19.2 | | | 17.8 | | 16.7 | 18.9 | 28.4 | | 27.2 | 29.6 | 23.8 | | | 21.9 | | 25.7 | | 21.6 | | | 20.5 | 22.7 | 20.4 | | | 19.5 | 21.4 | | 32.4 | | 29.5 | 35.2 |
| **2016/17** | 23.3 | 22.2 | 24.4 | 27.7 | | 26.3 | 29.1 | 17.9 | | 16.5 | 19.4 | | | 18.5 | | 17.4 | 19.6 | 27.9 | | 26.8 | 29.1 | 24.6 | | | 22.6 | | 26.5 | | 21.4 | | | 20.4 | 22.5 | 20.1 | | | 19.1 | 21.0 | | 33.1 | | 30.5 | 35.7 |
| **Change^ab^** | 50.3 | | | 55.6 | | | | 42.1 | | | | | | 65.2 | | | | 41.6 | | | | 51.9 | | | | | | | 52.9 | | | | | 43.6 | | | | | | 47.1 | | | |
| ^a^Percentage change; ^b^Adjusted test of trend for time, 2006/7-2016/17, p< 0.001 for all. | | | | | | | | | | | | | | | | | | | | | | | | | | | | | | | | | | | | | | | | | | | |
