# Supplementary material for: Physical fitness disparities among New York City public school youth using standardized methods, 2006-2017
Source: PLoS One. 2020 Apr 9;15(4):e0227185. doi: 10.1371/journal.pone.0227185 (PMC7144992; doi:10.1371/journal.pone.0227185)
Supplement: S2 Table — (DOCX) [file pone.0227185.s003.docx]

|  | Black Female | | | | Black Male | | | | Hispanic Female | | | Hispanic Male | | | | White Female | | | | | | White Male | | | Asian Female | | | | Asian Male | | | |
| --- | --- | --- | --- | --- | --- | --- | --- | --- | --- | --- | --- | --- | --- | --- | --- | --- | --- | --- | --- | --- | --- | --- | --- | --- | --- | --- | --- | --- | --- | --- | --- | --- |
| **Year** | % | 95% CI | | | % | 95% CI | | | % | 95% CI | | % | | 95% CI | | % | | | 95% CI | | | % | 95% CI | | % | | 95% CI | | % | | 95% CI | |
| **2006/07** | 10.1 | 8.7 | 11.5 | | 18.0 | 15.6 | 20.4 | | 9.6 | 8.3 | 10.8 | 18.4 | | 16.2 | 20.6 | 17.6 | | 14.3 | | 20.9 | | 27.0 | 23.8 | 30.1 | 12.1 | | 9.8 | 14.4 | 19.9 | | 17.3 | 22.5 |
| **2007/08** | 9.2 | 8.1 | 10.2 | | 17.7 | 16.0 | 19.5 | | 8.6 | 7.5 | 9.6 | 17.5 | | 16.0 | 19.0 | 17.4 | | 14.2 | | 20.6 | | 25.3 | 22.7 | 27.8 | 12.4 | | 10.4 | 14.4 | 19.4 | | 17.5 | 21.3 |
| **2008/09** | 10.2 | 9.1 | 11.3 | | 19.6 | 18.2 | 20.9 | | 9.3 | 8.4 | 10.1 | 18.3 | | 17.1 | 19.5 | 18.9 | | 15.9 | | 21.9 | | 28.0 | 25.1 | 30.8 | 13.4 | | 11.6 | 15.2 | 21.9 | | 19.8 | 23.9 |
| **2009/10** | 10.2 | 9.2 | 11.2 | | 20.8 | 19.4 | 22.1 | | 10.0 | 9.1 | 10.8 | 19.5 | | 18.4 | 20.6 | 19.4 | | 16.2 | | 22.5 | | 28.6 | 25.6 | 31.5 | 14.1 | | 12.2 | 16.0 | 23.7 | | 21.5 | 26.0 |
| **2010/11** | 10.8 | 9.7 | 11.9 | | 21.7 | 20.2 | 23.1 | | 10.5 | 9.6 | 11.3 | 21.0 | | 19.9 | 22.1 | 20.4 | | 17.2 | | 23.5 | | 30.3 | 27.3 | 33.4 | 14.8 | | 12.7 | 16.9 | 25.2 | | 22.9 | 27.6 |
| **2011/12** | 15.8 | 13.6 | 18.0 | | 26.0 | 23.8 | 28.2 | | 11.6 | 10.4 | 12.8 | 22.6 | | 21.2 | 24.0 | 11.0 | | 10.1 | | 11.9 | | 21.9 | 20.8 | 23.0 | 21.6 | | 18.1 | 25.1 | 31.8 | | 28.8 | 34.9 |
| **2012/13** | 11.8 | 10.8 | 12.9 | | 23.9 | 22.5 | 25.2 | | 11.6 | 10.7 | 12.5 | 22.7 | | 21.6 | 23.9 | 22.3 | | 18.9 | | 25.7 | | 33.5 | 30.5 | 36.4 | 16.4 | | 14.2 | 18.6 | 26.5 | | 24.4 | 28.7 |
| **2013/14** | 13.2 | 12.2 | 14.2 | | 25.9 | 24.7 | 27.2 | | 12.5 | 11.7 | 13.4 | 23.7 | | 22.6 | 24.9 | 23.3 | | 20.5 | | 26.0 | | 35.3 | 32.5 | 38.2 | 17.5 | | 15.6 | 19.4 | 27.5 | | 25.4 | 29.6 |
| **2014/15** | 14.7 | 13.6 | 15.8 | | 26.9 | 25.6 | 28.2 | | 13.5 | 12.7 | 14.4 | 24.5 | | 23.4 | 25.6 | 24.0 | | 21.3 | | 26.6 | | 36.0 | 33.2 | 38.7 | 18.5 | | 16.5 | 20.5 | 27.3 | | 25.1 | 29.5 |
| **2015/16** | 15.7 | 14.6 | 16.8 | | 27.4 | 26.1 | 28.7 | | 15.0 | 14.1 | 16.0 | 25.6 | | 24.5 | 26.7 | 26.9 | | 24.0 | | 29.9 | | 37.5 | 34.6 | 40.3 | 19.3 | | 17.3 | 21.2 | 28.1 | | 26.0 | 30.2 |
| **2016/17** | 16.2 | 15.1 | 17.2 | | 26.7 | 25.4 | 27.9 | | 15.3 | 14.3 | 16.2 | 24.6 | | 23.6 | 25.7 | 28.3 | | 25.6 | | 31.1 | | 37.6 | 34.9 | 40.2 | 20.4 | | 18.5 | 22.3 | 28.4 | | 26.2 | 30.6 |
| **Change^ab^** | 60.4 | | | 48.3 | | | | 59.4 | | | | | 33.7 | | | | 60.8 | | | | 39.3 | | | | | 68.6 | | | | 42.7 | | |
| ^a^Percentage change; ^b^Adjusted test of trend for time, 2006/7-2016/17, p< 0.001 for all. | | | | | | | | | | | | | | | | | | | | | | | | | | | | | | | | |
